# Study on the Relationship between Seed Absorbed Dose and Seed Composition of ^252^Cf Neutron Source Irradiated Bean Seed

**DOI:** 10.1038/s41598-019-45829-1

**Published:** 2019-07-03

**Authors:** Dapeng Xu, Yingguo Li, Ze’en Yao, Yongzhi Yin, Huyuan Feng, Zheng Wei

**Affiliations:** 1 School of Nuclear Science and Technology, Lanzhou University, Lanzhou, 730000 China; 2 School of Life Sciences, Lanzhou University, Lanzhou, 730000 China; 3 Engineering Research Center for Neutron Application Technology, Ministry of Education, Lanzhou University, Lanzhou, 730000 China

**Keywords:** Agricultural genetics, Characterization and analytical techniques

## Abstract

This study aims to further identify the biological effects of neutron-irradiated plants and provides insights into the mutation breeding of such plants. In this study, the neutron irradiation device designed by our institute was used to analyze the relationship between the seed components in different legume crops and their neutron absorption dose rate, fission gamma absorption dose rate, and induced gamma absorption dose rate. The results show that the effect sizes of the components on the neutron absorbed dose rate are as follows: ash > fat > moisture > carbohydrate > protein. The effect sizes of the components on the absorbed dose rate of fission gamma are as follows: ash > moisture > fat > carbohydrate > protein. There is a positive correlation between fission gamma absorbed dose rate and the weight of ash, water and fat, while a negative correlation with carbohydrate and protein. However, the linear relationship between each component and the absorbed dose rate of induced gamma is not significant, this needs to be identified by further researches. Based on the results of the present study, we conclude that the neutron absorbed dose can be calculated without taking into account the fat composition of bean crop seeds (except for soybean seeds) in the process of mutation breeding induced by radiation. In special cases where the accuracy requirement of the dose rate is not high, it is possible to use protein instead of legume crop seeds for neutron absorption dose calculations.

## Introduction

It has been 80 years, since the botanist, Stadler, proved the X-ray irradiate barley can cause mutagenesis effect^1^. In the past time, scientists have irradiated different plants with various rays, making great achievements in radiation biological effects and mutation breeding, and accumulating rich research data (eg, literature)^2–10^. Neutron, discovered by Chadwich *et al*.^11^, is a high LET (Linear Energy Transfer) ray, which has the characteristics of strong penetration, wide variation spectrum, high variation rate, and relatively stable character of variant offspring. Compared with γ-ray photons, Neutron can produce more obvious and higher radiation biological effect and attracted much attention from researchers (eg, literature)^7–10^.

To carry out the research work on biological effects and mutation breeding of neutron radiation plants, we designed an experimental device for neutron irradiated plant seeds based on ^252^Cf source. The irradiation device has the characteristics of convenient sample placement and high uniformity of absorbed dose in each irradiation area. The correlation between the content of different components in seeds and their absorbed dose rate was studied for the neutron irradiated seeds of bean plants. (Terms: Neutron absorbed dose rate refers to neutron energy deposition per unit time per unit mass sample.Fission gamma absorbed dose rate represents the fission gamma ray energy deposition per unit time per unit mass sample. Induced gamma absorbed dose rate is the energy deposition of induced gamma ray per unit of time and mass in the sample).

## Methods

### Bean crops and their seed components

Bean crops refer to the general name of leguminous crops, mainly including soybeans, broad beans, peas, mung beans, and kidney beans. The main chemical components of the bean seed include protein, carbohydrate (containing cellulose), fat, water and ash. The mass percentages of the five components are: 21–37%, 50–61%, 1–3% (except soybean seeds), 7–12% and 2–7%^12^, respectively. The proportion of four elements of C, H, O and N in protein is more than 97%; carbohydrate (containing cellulose) and fat contain three elements: C, H, O; and only two elements H and O are contained in water. We measured the elements contained in the ash of pea. The results show that the content of the four elements, K, P, Mg, Ca, were the highest, accounting for about 90% of the total ash content.

### Selection of methods for obtaining absorbed dose rate

**Figure 1:**
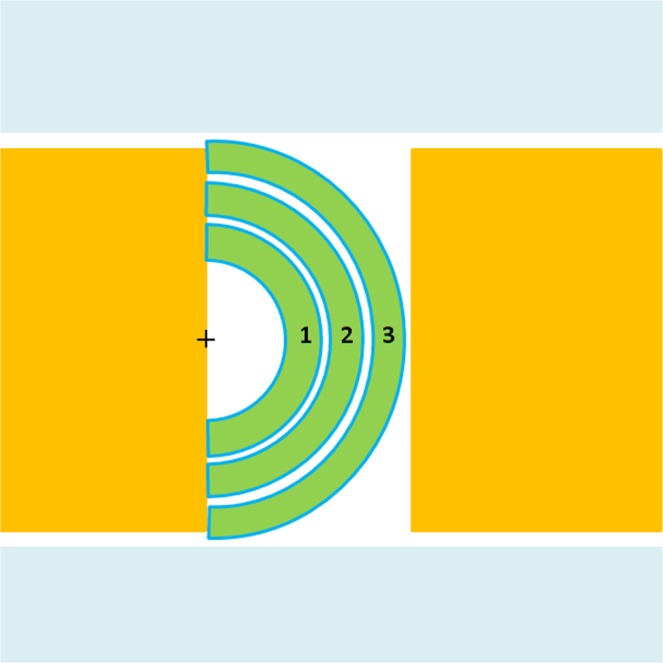
Illustriation of the three zones in the Neutron radiation device.

Figure 1 is a schematic diagram of the irradiation sub-area of the device designed. The black “+” symbol in the figure is a ^252^Cf neutron source. The main irradiation area is divided into three irradiation zones: Zone 1, Zone 2 and Zone 3. The diameter of the seed samples in the irradiation zones is 7 mm. Due to the large number of samples per sub-zone when irradiating seed samples, it is not possible to measure the absorbed dose rate of each seed experimentally. Therefore, it is necessary to use Monte Carlo’s method, MCNP program^13^, to simulate the absorbed dose rate of each zone. Based on the literature^14^ and our previous simulation verification, we decided to use the *F6 card to directly measure the deposition energy count in each sub-area. The neutron and gamma absorbed dose rates of the seeds in the corresponding subarea are given after the unit is converted.

### The modeling process of seed composition

The reference percentages of the five components in the bean seeds are: 5% ash, 9% moisture, 29% protein, 55% carbohydrate (cellulose-containing), 2% fat. The mass ratio of the five components is ash: Moisture: Protein: carbohydrate (cellulose-containing): Fat = 5: 9: 29: 55: 2. Except for the variable components examined, the mass ratio between the other four components will maintain the above proportional relationship in the simulation process. The mass percentages of the investigated variables was taken as: ash 0–7%, water 7–12%, protein 21–37%, carbohydrate (containing cellulose) 50–61%, and fat 0–3%, respectively.

## Results and Discussions

### The relationship between ash content and absorbed dose rate

**Figure 2:**
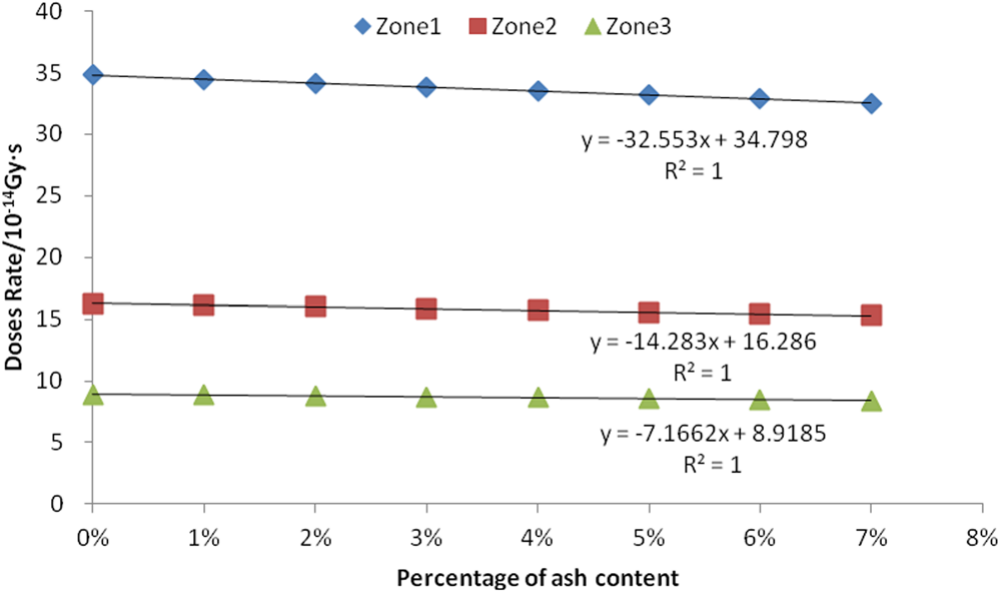
Relationship between seed ash content and neutron absorbed dose rate.

**Figure 3:**
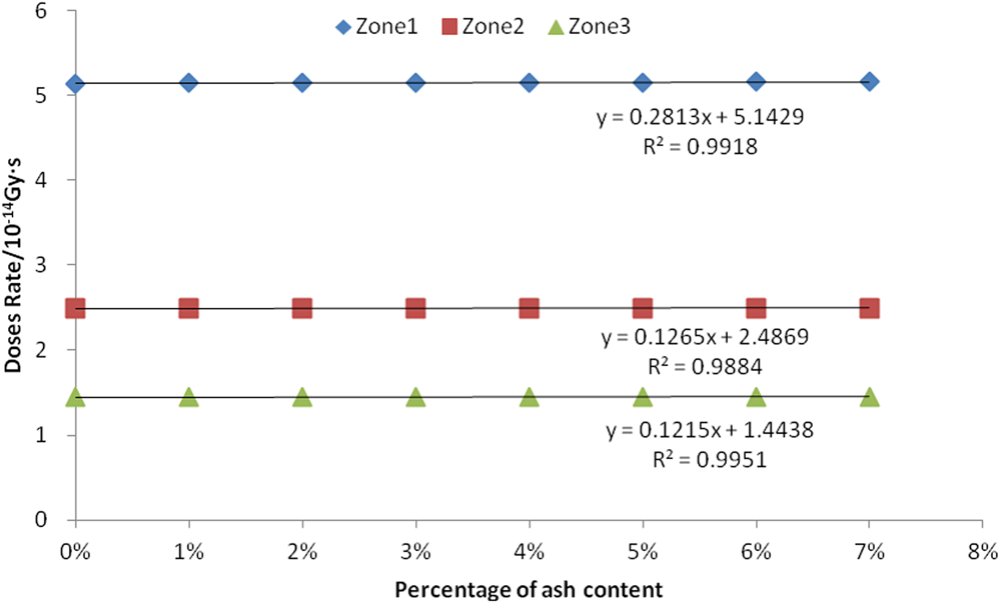
Relationship between ash content and fission gamma absorbed dose rate.

We calculated the eight consecutive values of the ash content percentage in the bean seeds. The eight values are 0%, 1%, 2%, 3%, 4%, 5%, 6% and 7%. After calculating the absorbed dose rates of neutrons, fission gamma and induced gamma in the three sub-regions by using simulated data, the linear correlation analysis is performed, as shown in Figs 2 and 3 and Table 1.

**Table 1:** Linear relationship between ash content and induced gamma absorbed dose rate.

Irradiation zones	Linear regression equation y = ax + b	Determination coefficient R^2^	Significance P
Zone 1	y = 0.1507x + 0.2455	0.9833	0.000
Zone 2	y = 0.1446x + 0.2433	0.9948	0.000
Zone 3	y = 0.1169x + 0.2446	0.9977	0.000

The results showed a negative correlation between the mass percentage of seed ash and the neutron absorbed dose rate. The R^2^ values of the corresponding three linear equations in all three zones are close to 1, indicating that the fit is very linear. The P value of the F-test is 0.000, indicating that this negative correlation is extremely significant. There was a positive correlation between the mass percentage of seed ash and absorption dose rate of both fission gamma and the induced gamma in the three zones. The R^2^ values are all greater than 0.98, indicating that the equation is also very good in terms of goodness of fit. The F-tested P-values are all 0.000, indicating that this positive correlation is extremely significant.

### Relationship between moisture content and absorbed dose rate

During the simulation, six consecutive percent moisture content of bean seeds were selected. These six values were 7%, 8%, 9%, 10%, 11%, and 12%. When the simulation was completed, the absorbed dose rates for neutrons, fission gammas, and induced gammas were calculated in the three zones. The linear correlation analysis was also performed.

**Figure 4:**
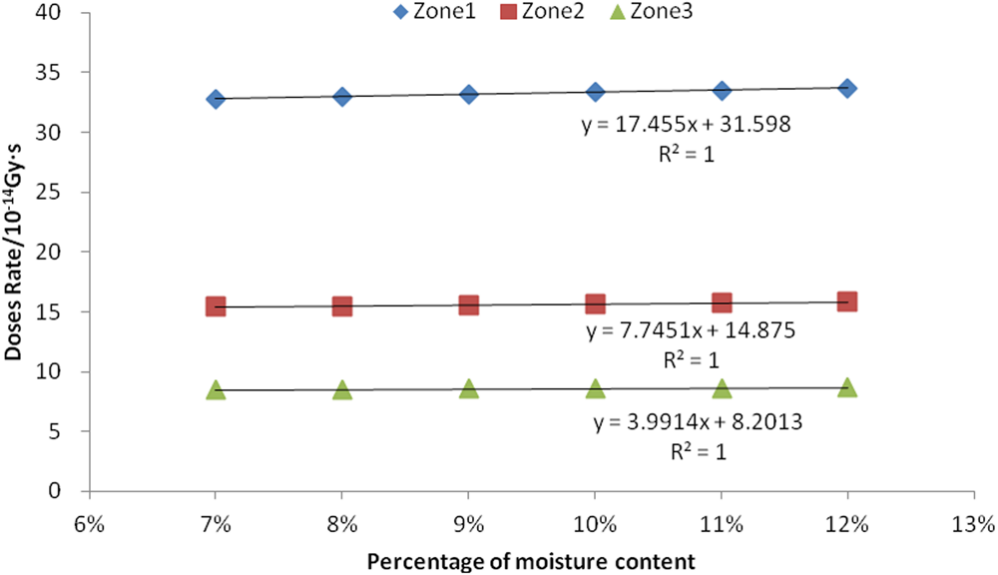
Relation between seed moisture content and neutron absorbed dose rate.

**Figure 5:**
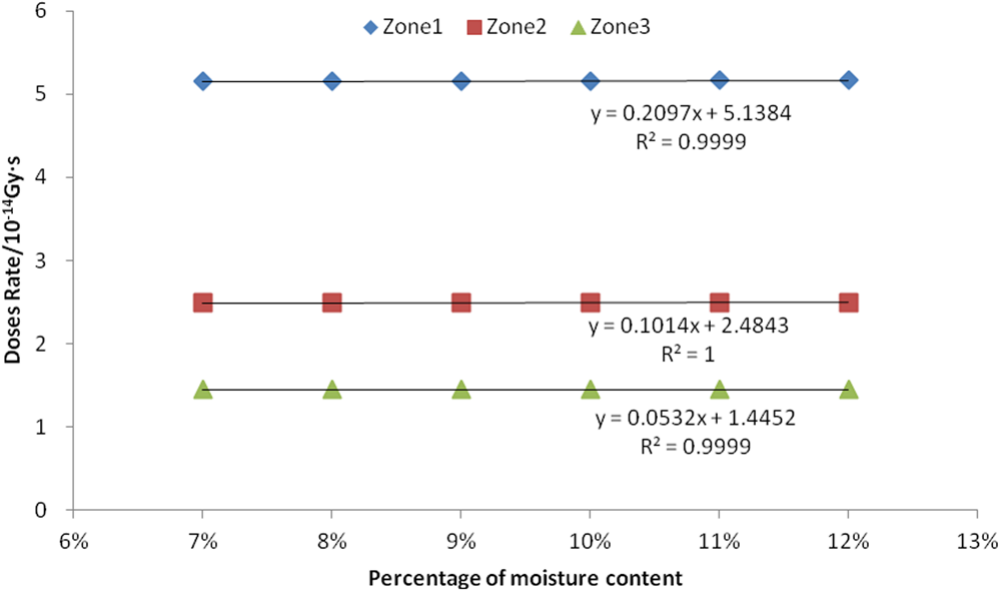
Relation between seed moisture content and fission gamma absorbed dose rate.

The results are shown in Figs 4 and 5, and Table 2. From Figs 4 and 5, it can be seen that there is a positive correlation between the mass percentage of seed moisture and absorbed dose rate of both neutron and fission gamma in the three zones. The R^2^ values of the corresponding six linear equations are all greater than 0.999, indicating that the fitted equations are very linear. The P values of the F-test are 0.000, indicating that this positive correlation is extremely significant. It can be seen from Table 2 that there is a positive correlation between the mass percentage of seed moisture in zone 2 and zone 3 and the induced gamma absorption dose rate. Both the corresponding two linear equation R^2^ values are greater than 0.94, indicating that the linear equation is also very good in fitting, and the F-test P-value results are less than 0.002, indicating that this positive correlation is extremely significant. In addition, the linear equation fit between the mass percentage of seed moisture in zone 1 and the induced gamma absorption dose rate was found to be very low and not significant, and therefore it was judged that they had no close correlation.

**Table 2:** Linear relationship between seed moisture content and induced gamma absorption dose rate.

Irradiation zones	Linear regression equation y = ax + b	Judgment coefficient R^2^	Significant P
Zone 1	y = 0.0101x + 0.2525	0.297	0.263
Zone 2	y = 0.0297x + 0.248	0.9711	0.000
Zone 3	y = 0.0273x + 0.2481	0.9486	0.001

### The relationship between protein content and absorbed dose rate

**Figure 6:**
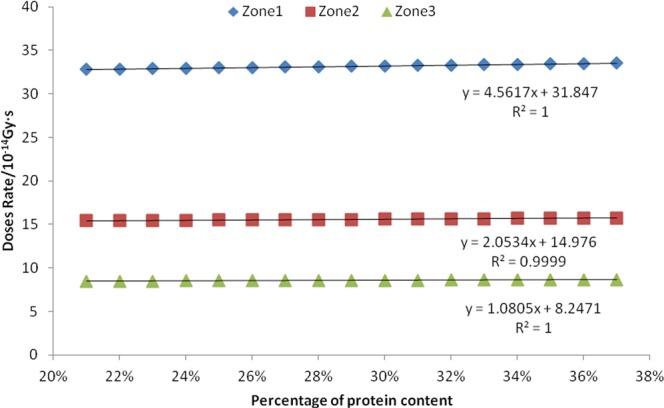
Relationship between seed protein content and neutron absorption dose rate.

**Figure 7:**
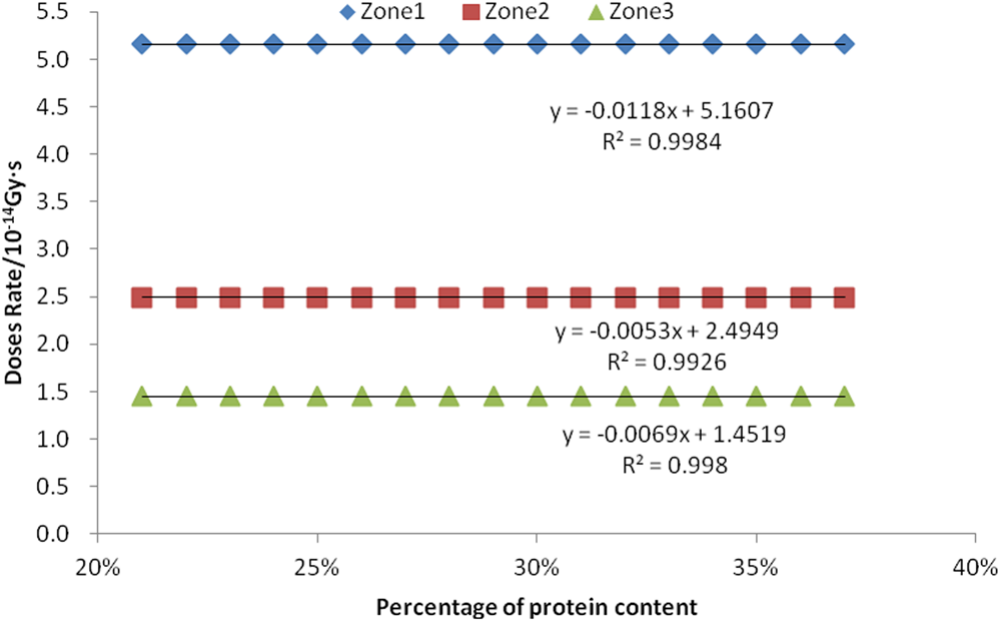
Relationship between seed protein content and fission gamma absorption dose rate.

We selected seventeen consecutive values of mass percentage of seed protein as 21%, 22%, 23%, 24%, 25%, 26%, 27%, 28%, 29%, 30%, 31%, 32%, 33%, 34%, 35%, 36%, and 37%. For simulation data, the absorption dose rates of neutrons, fission γ, and induced γ in the three sub-areas were obtained, and then a linear correlation analysis was performed, as shown in Figs 6 and 7, and Table 3.

**Table 3:** Linear relationship between seed protein content and the induced gamma absorption dose rate.

Irradiation zones	Linear regression equation y = a x + b	Judgment coefficient R^2^	Significant P
Zone 1	y = −0.0086x + 0.2558	0.5225	0.019
Zone 2	y = −0.0063x + 0.2526	0.866	0.001
Zone 3	y = −0.0066x + 0.2525	0.8952	0.000

Figure 6 shows that there is a positive correlation between the neutrons absorbed dose rate and the mass percentage of protein in the seeds in the three zones. The R^2^ values of the corresponding three linear equations are all greater than 0.999, indicating that the fitted equations are very linear. The P value of the F-test is 0.000, indicating that this positive correlation is extremely significant. There is a negative correlation between the mass percentage of seed protein and the absorption dose rate of fission gammas and induced gammas in the three zones. The R^2^ values of the fission γ of three linear equations are greater than 0.99. R^2^ values of linear equations for the induced γ in zone 2 and zone 3 are greater than 0.86. However, the R^2^ value of the zone 1 linear equation for induced gamma is 0.5225, and the P value obtained by F-test is 0.019, which indicates that the positive correlation is only general significance, indicating that the induced gamma absorbed dose rate in the zone 1 has a low correlation with the mass percentages of proteins.

### The relationship between carbohydrate content and absorbed dose rate

Twelve consecutive values of the carbohydrate content percentage in bean seeds selected during the simulation were 50%, 51%, 52%, 53%, 54%, 55%, 56%, 57%, 58%, 59%, 60% and 61%. The absorbed dose rates for neutrons, fission γ, and induced γ were calculated for all three zones, and linear correlation analysis were calculated.

**Figure 8:**
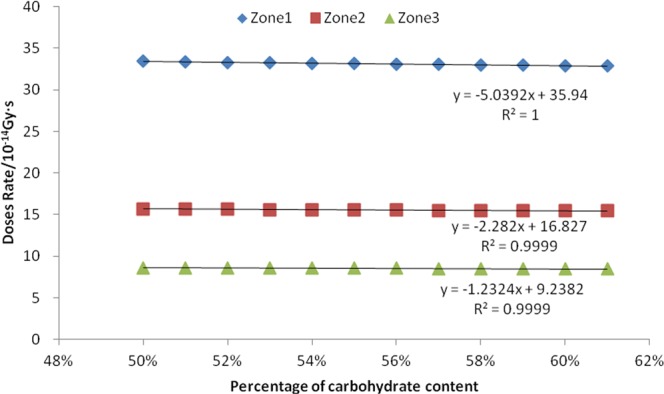
Relationship between seed carbohydrate content and neutron absorption dose rate.

**Figure 9:**
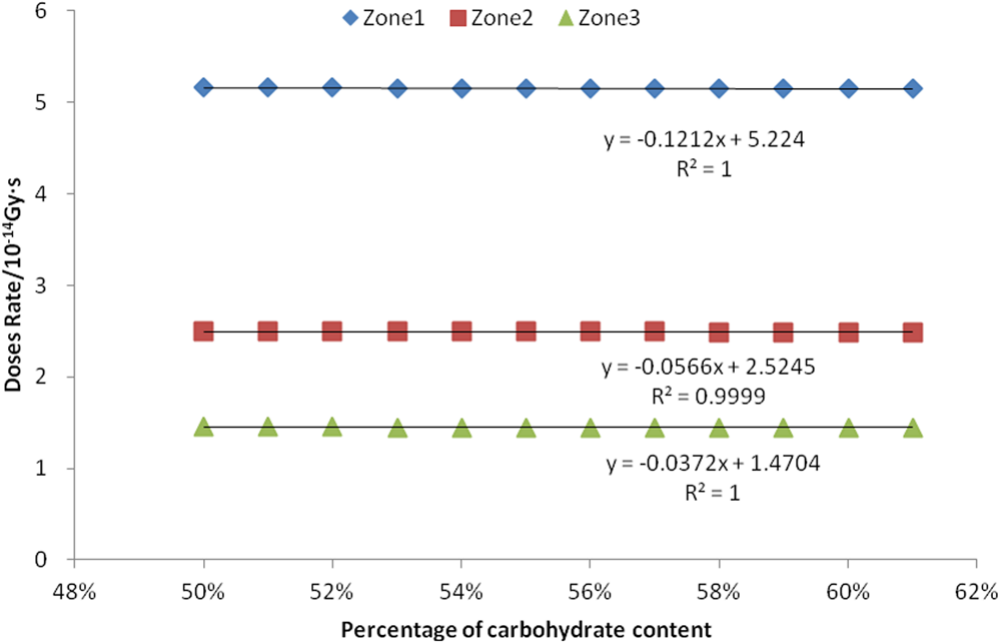
Relationship between seed carbohydrate content and fission gamma absorption dose rate.

It can be seen from Figs 8 and 9 and Table 4 that there is a negative correlation between the mass percentage of seed carbohydrate and the absorbed dose rate for neutrons, fission gammas, and the induced gammas. The R^2^ values of the linear equations in the corresponding three zones are all greater than 0.95, indicating that the fitted equations are very linear. The result of the F-test P-value is 0.000, indicating that this negative correlation is extremely significant.

**Table 4:** Linear relationship between seed carbohydrate content and induced gamma absorption dose rate.

Irradiation zones	Linear regression equation y = a x + b	Judgment coefficient R^2^	Significant P
Zone 1	y = −0.0377x + 0.2743	0.9596	0.000
Zone 2	y = −0.032x + 0.2684	0.9914	0.000
Zone 3	y = −0.0272x + 0.2655	0.9881	0.000

### Relationship between fat content and absorbed dose rate

**Figure 10:**
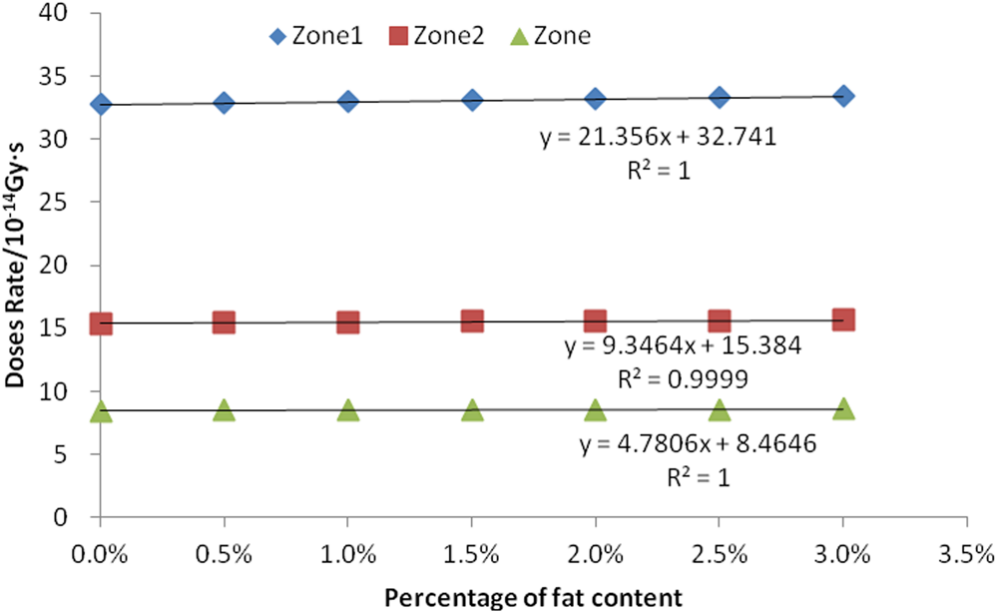
Relationship between seed fat content and neutron absorption dose rate.

**Figure 11:**
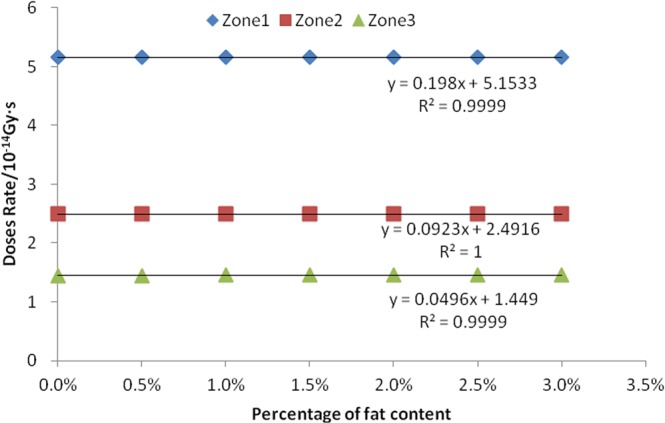
Relationship between seed fat content and fission gamma absorption dose rate.

Our simulation was based on the seven consecutive values of the fat percentage of legume seeds. The seven values were 0%, 0.5%, 1%, 1.5%, 2%, 2.5%, and 3%. The linear correlation analysis was performed after calculating the absorption dose rates of neutrons, fission γ, and induced γ in the three zones, as shown in Figs 10 and 11, and Table 5.

**Table 5:** Linear relationship between seed fat content and induced gamma absorption dose rate.

Irradiation zones	Linear regression equation y = a x + b	Judgment coefficient R^2^	Significant P
Zone 1	y = 0.0444x + 0.2527	0.6093	0.038
Zone 2	y = 0.0293x + 0.2502	0.9328	0.000
Zone 3	y = 0.0234x + 0.25	0.9106	0.001

The results show that there is a positive correlation between the mass percentage of seed fat and the absorbed dose rate of neutrons, fission gammas, and the induced gammas in the three sub-regions. The R^2^ values of the linear equations are all greater than 0.91 for neutrons and fission gammas in three zones, and for the induced gammas in zone 2 and zone 3. This indicates that the fitted equations are very linear. The result of P value is less than 0.001, indicating that this positive correlation is extremely significant. However, the R^2^ value of the linear equation of induced γ in zone 1 is 0.6093, and the result of F-test is 0.038, which indicates that the positive correlation is only of general significance. The correlation between the γ absorbed dose rate and its fat mass percentages is low in zone 1.

### Discussions and analysis

We found that the effect of various components on the neutron absorption dose rate is: Ash > Fat > Moisture > Carbohydrate > Protein. There is a positive correlation between the mass percentages of fat, moisture, and protein and the neutron absorbed dose rate. For ash and carbohydrate, there is a negative correlation between neutron absorbed dose rate and the mass percentages. The effects of various components on the fission gamma absorption dose rate are as follows: Ash > Moisture > Fat > Carbohydrate > Protein, where there is a positive correlation between ash, moisture, and fat and neutron absorption dose rate. However, there is a negative correlation between the neutron absorption dose rate and carbohydrates and proteins. For the ^252^Cf neutron source, the fission gamma yield is 5.67 times than that of the neutron yield. The neutron absorption dose rate for the single-particle irradiation of the three sub-regions obtained is 5.7–6.8 times than that of the fission gamma absorption dose rate. In the ^252^Cf neutron source irradiation experiment, the neutron absorption dose was slightly greater than the fission gamma absorption dose. In addition, during the study of radiation biological effects, it has been confirmed that the biological effect of neutron radiation is tens of times of the biological effects of gamma radiation^15^. Therefore, it is necessary to further analyze the underlying causes of neutron absorbed dose rate. Table 6 shows the correlation between the changes of the corresponding eight elements and the neutron absorbed dose rate when the different components in Part 1 were changed. We found that regardless of the compositional change, only the change in the H element and the dose rate remained positively correlated. This shows that H element is the main factor affecting the neutron absorption dose rate.

**Table 6:** Correlation between the different components and corresponding elements and neutron absorbed dose rate in zone 1.

components	Linear dependence of different components on dose rate	Correlation of the elements effect on dose rate							
Ash	negative	C	H	O	N	K	P	Mg	Ca
		P	P	P	P	N	N	N	N
Moisture	positive	C	H	O	N	K	P	Mg	Ca
		N	P	P	N	N	N	N	N
protein	positive	C	H	O	N	K	P	Mg	Ca
		P	P	N	P	N	N	N	N
carbohydrate	negative	C	H	O	N	K	P	Mg	Ca
		N	P	N	P	P	P	P	P
fat	negative	C	H	O	N	K	P	Mg	Ca
		P	P	N	N	N	N	N	N

Table 7 shows the ratio of the minimum to maximum neutron absorption doses in each subarea within the range of the content of each component of legume crop seeds. It was found that the ratio of fat was closest to 1, and the main reason was that the range of fat content was the smallest. The ratio of protein in the mass percentage range of 17% is very close to 1, which can be found from the smaller slope of the linear equation in Fig. 6. In the process of radiation induced mutation breeding, the neutron absorbed dose can be calculated without considering the fat composition of legume crop seeds (except soybean). In addition, in some cases where the accuracy of the dose rate requirement is not high or in exceptional cases, protein can be used instead of legume crop seeds to calculate neutron absorbed doses.

**Table 7:** Ratio of neutron absorbed dose minimum and maximum values in different composition variation range.

Irradiation zones	Ash D_min_/D_max_	Moisture D_min_/D_max_	Protein D_min_/D_max_	carbohydrate D_min_/D_max_	fat D_min_/D_max_
Zone 1	0.9524	0.9741	0.9783	0.9834	0.9872
Zone 2	0.9553	0.9755	0.9792	0.9840	0.9880
Zone 3	0.9591	0.9770	0.9800	0.9842	0.9889

## Summaries

This study found a significant linear relationship between the composition of legume crops seeds and its neutron absorbed dose rate, as well as fission gamma absorbed dose rate. The significance of linear relationship between the composition of legume crops seeds and the induced gamma absorbed dose rate of is not very high, especially in the zone 1 this linear correlation phenomenon cannot be judged. The main reason is that the induced gamma was produced by the interaction between neutrons and materials. The pea seeds in the whole irradiation area become gamma sources, this increased the complexity. Zone 1 is the nearest area to neutron source in the whole irradiation area, which is the reason for the worst linear correlation in zone 1. Fortunately, the dose rate of induced gamma is one order of magnitude lower than that of fission gamma, which may not be taken into account in the current radiation biology research. With the exploration of the mechanism of radiation mutagenesis, the further research and analysis are still needed.
